# Organic Creativity for Well-Being in the Post-Information Society

**DOI:** 10.5964/ejop.v13i4.1547

**Published:** 2017-11-30

**Authors:** Giovanni Emanuele Corazza

**Affiliations:** a Department of Electrical, Electronic, and Information Engineering "Guglielmo Marconi", Marconi Institute for Creativity, University of Bologna, Bologna, Italy

**Keywords:** creativity, industrial society, information society, post-information society

## Abstract

The editorial dwells upon the technology-driven evolution from the Industrial to the Post-Information Society, indicating that this transition will bring about drastic transformations in our way of living, starting from the job market and then pervading all aspects at both individual and social levels. Great opportunities will come together with unprecedented challenges to living as we have always known it. In this innovation-filled scenario, it is argued that human creativity becomes the distinctive ability to provide dignity at first and survival in the long term. The term organic creativity is introduced to indicate those conditions, attitudes, and actions that bear the potential to be at the same time productive in socio-economic terms and conducive to human well-being. As a consequence, the role of psychologists in an open cooperation with sociologists, economists, computer scientists, engineers and others, will be as central as ever in establishing healthy collaboration modes between humans and machines, and large investments in related multidisciplinary scientific research are advocated to establish organic creativity as a discipline that should permeate every educational level, as well as our professional and everyday lives.

When my friend and EJOP Editor Vlad Glăveanu asked me to write an editorial message to the Journal’s readership, I felt both very honored and challenged in my responsibility to provide you with what I consider to be critical future-relevant information, from a sort of an insider’s point of view. Indeed, my intellectual position can be considered to be rather peculiar: an electronic engineer who built his academic career on the development of mobile telephony and satellite communications, and who in 2011 founded an institute dedicated to human creativity studies in the name of the inventor of radio, the Marconi Institute for Creativity (http://mic.fgm.it). It is from this quite atypical viewpoint that I want to anticipate here my main conclusion: the role of psychology in the future Post-Information Society will be as fundamental as ever, given the unprecedented challenges and opportunities for the well-being of humanity, and it should be in the driving seat of a multidisciplinary endeavor involving as a minimum sociology, economy, computer science and engineering. Increased investments in creativity research and applications are urgently necessary, in order to provide answers to fundamental questions that are both scientifically exciting and socially troublesome. In the development that leads to this conclusion, interesting resonance will be found with the two preceding EJOP editorials by Vlad Glăveanu himself (Glăveanu, 2017) and James Kaufman (Kaufman, 2017), thus forming a sort of continued discussion flow, threaded by the common fil rouge of identifying major endeavors for the benefit of humanity.

I happened to start writing this editorial while travelling in Moscow, where I was invited to speak at the Open Innovations Forum, at the Skolkovo Technology Park. There, the latest developments in artificial intelligence, robotics, nano-materials, digital infrastructures and services were presented to a very large audience, in a sort of positivistic celebration of the advent of new technologies, along the inevitable path towards the Post-Information Society. And, to be honest, the technological progress that we are witnessing is nothing less than impressive, in both substance and pace. It is not only a question of improving the performance of technologies we already know, but the introduction of completely new devices, solutions, and systems that have the potential to revolutionize all sectors in society, including healthcare, transportation, manufacturing, management, and entertainment, just to name a few. One could think that this will be yet another industrial revolution, as our species has witnessed since the eighteenth century, and that we have collectively proven our capacity to live through these periods of transition, notwithstanding the inherent challenges. However, this specific phase of technological progress appears to be different from all preceding ones, in that it sees the actual come into play of a new form of machine cognition, namely artificial intelligence; therefore, the interconnection between technology, human cognition and human life in general will become much more intrinsic, invisible, and impactful. Clearly, artificial intelligence is not a new concept (see for a brief review the historical notes in Simon, 1995); but now its level of maturity has overcome the threshold to really enter into our lives and affect them, and this proximity was indeed tangible at the Open Innovations Forum. To give you a sense of the atmosphere, I attended a session entitled: “*Smarter than others. How humans are being replaced by Artificial Intelligence in corporations*”. Capital letters “*A*”, “*I*” not added by the Author. It would have taken the panelists only a slight detachment from their love for technology advances to realize that the numbers they were presenting were dramatic in their potential impact. In a nutshell: while in the history of the Industrial Society the curves for productivity and employment always grew hand-in-hand as a function of time, since the start of the third millennium we have been facing a growing separation, which in the press has been identified as the “decoupling” (Brynjolfsson & McAfee, 2012). Decoupling entails that a direct proportionality between economic growth and population well-being cannot be taken for granted, anymore: productivity can continue or accelerate its growth while at the same time employment can stagnate or decline. The debate about the extent and the motivations behind this decoupling is today certainly open and non-obvious (in fact, it might easily become another politically charged argument, in some way similar to climate change discussions), but in any case there is no doubt that the new technologies are playing a key role in transforming industrial economy and the associated job market. To report an example: automated driving and self-driving cars, which are forecast to become a widespread reality in the next ten to fifteen years, producing an exciting estimated market in the order of 2 to 7 trillion dollars, bear the potential to produce a massive earthquake in the job market. In fact, approximately 10% of human jobs today involve operating a vehicle. The balance between lost professions and the introduction of new ones is a hot subject for open debates, involving economic, social, and political interests. But while the most immediate consequences of these transformations are visible in economy and specifically in the job market, the social and psychological implications are just behind the corner. Indeed, at the Open Innovations Forum the most recurring question from the audience was: “And what about the role of humans, given this abundance of new very efficient and smart technologies?” Replies from speakers took on different forms of what could be defined as a smiling embarrassment. Clearly, none of the presenters had really given serious thought about the psychological and social impact of these transformations, simply because it was not their job. One fact should be very clear: this technological progress cannot be stopped or even slowed down. Humans will transform society through machines and will have to learn to live well with them. Therefore, it is imperative that we understand and possibly anticipate the foreseeable consequences, the opportunities as well as the threats that will be offered, and that we parallel technological developments with urgent work on the human side to enable new forms of well-being in the Post-Information Society. The pace of transformation is simply too fast to wait for natural adjustments, reactions, and retroactions: radical change will occur in less than a generation.

Let’s enter into a few analytical observations. Whereas the Industrial Society was based on standardization of products and concentration of resources, the Information Society came in as a technological revolution through personalization and distribution (Corazza, Vanelli-Coralli, & Pedone, 2010), leading to the complete de-structuring of space and time, such that we can live, work, and interact almost irrespectively of the physical site in which we are at the present moment. It should be noted that this does not mean that cognition is disembodied, but rather that we are witnessing manifold embodiment into diversified physical and virtual realities. As a consequence of the introduction of distributed databases in cloud-based infrastructures, information has become a commodity, at the disposal of anyone with access to the Internet and its search engines, that can actually be considered to be the first pervasive form of non-anthropomorphic artificial intelligence. These changes have raised a first set of proximal questions at the individual and relational levels, that invest the realms of psychology, sociology, anthropology, and education. Many of these questions are presently under investigation (e.g., see Loh & Kanai, 2016, and the references therein), but definitive answers will of course require longer longitudinal efforts. How many times per day do we search the Internet to find out facts that we ignore, or more basically to recall things that we seem to have forgotten? What are the effects of this daily behavior on our long-term memory and deep-learning skills? In particular, what will be the impact on the cognitive processes and abilities of a native digital, who cannot even imagine a world without a search engine? What are the effects on critical thinking skills and reflexivity, the lack of which could also lead to the rise of forms of post-truths, as hypothesized in the insightful editorial by Vlad Glăveanu (2017)? How should the education system be modified, given the pervasive knowledge connectivity provided by the Information Society? And finally a fundamental question: if a non-anthropomorphic form of artificial intelligence can in the limit provide all information to everyone instantaneously, what will be the key to make a difference between the endeavors of human beings, at both professional and everyday levels? As we have argued in (Corazza, 2016), these characteristics of the Information Society bring as a consequence that it is virtually impossible to build a walled garden around one’s knowledge; therefore, the most and perhaps only distinctive behavior of a human being becomes *creativity*, exemplified as taking elements from the shared layer of information to generate ideas and actions bearing a potential to be both original and effective with respect to one’s goal. Diversity will not reside anymore in know-how, but in the ability to generate outcomes that are not yet known to (and possibly out of reach of) the non-anthropomorphic form of artificial intelligence surrounding us. Given these conditions, creativity cannot be a luxury for a few “geniuses” as was the case in the Industrial Society and throughout the preceding history, or a characteristic of outliers living at the margins of society, but it becomes a democratic necessity for everyone. As discussed in the passionate autobiographic editorial by James Kaufman (2017), in this view it is a must to move away from a stereotypical view of creativity as related to forms of mental illness, or in any case to variants of non-belonging, and concentrate our efforts on what can be identified as *organic creativity*, defined as the *potential for originality and effectiveness conducive to personal and social well-being*. This definition builds on the dynamic definition for creativity (Corazza, 2016) adding the general requirement of interrelationship with human health and happiness. An important note should be made here: it is evident that well-being can and should continue to be pursued in many alternative ways, that not necessarily involve any form of creative behavior. However, it is very important to underline that the critical peculiarity implied by organic creativity is that the pursuit of happiness is joint with a productive behavior, intended to exploit all of the potential benefits provided by the technological assets of the Information Society, and not at all detached from it. Once established, this approach will call for new forms of psychological and social intervention. Clearly, these are very ambitious goals, clarifying and pursuing which will require our studies to be organized into the form of a scientific discipline, striving to identify principles, traits, abilities, strategies and tools for practical application of organic creativity into everyday and professional lives. The central role of psychology in this endeavor cannot be overestimated: human dignity itself depends on creative behavior in the Information Society. Certainly, new technologies can be of help in this framework through fruitful collaborations between humans and machines, possibly contemplating also the virtues and power of computational creativity (Colton, de Mántaras, & Stock, 2009); but we should actively work to preserve and enhance the authentic, emotional, unique capacity of human minds to intentionally generate truly original and effective outcomes in our relational mesh leading to cultural accumulation. Authenticity is a fundamental element in establishing originality.

However, this is not the end of this editorial: while the above discussion might seem already to add up to a quite complex picture, it is still not sufficient to provide a medium/long-term look into our future. Whilst we are still adjusting to the networked environments created by the Information Society, the technological evolution continues with an accelerating pace, shortening considerably the time span available for personal, social, as well as political adjustment. In 2017, we start to see clearly that evolved forms of anthropomorphic and non-anthropomorphic artificially intelligent systems are maturing and will shortly become part of our world, bringing many proximal benefits but also unprecedented challenges. The Internet-of-Things will interconnect not only personal devices, but all objects, forming a huge cyber-mesh of human and non-human nodes, much more powerful and pervasive than the Internet we know today. As we enter into the Post-Information Society, characterized by the so-called second machine age (Brynjolfsson & McAfee, 2014), we should be aware of the fact that everything that happens on the Internet of Humans & Things, sensed and collected by applications running on personal or distributed devices, produces pieces of data, that accumulated on millions and billions of everyday instances generate the so-called Big Data and the consequent discipline of Data Science, intended as the extraction of useful information out of mines of data through sophisticated analytical methodologies. In fact, while jobs are being lost in traditional professions, this is clearly a direction of rapid growth in terms of employment; so much so that the “data scientist” has been indicated as the sexiest job of the 21st century (Patil & Davenport, 2012). In that article, the opening anecdote describes how the LinkedIn social network entered its real expansion trajectory thanks to the introduction of an artificially intelligent algorithm able to suggest “people you may know”, based on analyzing the user’s profile and his/her present network of relationships. In other words: an intelligent machine suggesting who your next connection could be. The list of Data Science applications is extremely long, and yet open to unforeseen breakthroughs. The following are but a few examples: sentiment analysis, adaptive marketing, automatic fraud detection, cyber-security algorithms, extracting patterns of behavior that enable personalized predictions about the networks of social relationships, DNA analysis that allows hyper-personalized medicine, as well as accurate lifetime span predictions by insurance companies. It is very important to maintain a balanced and wisely optimistic view on these advancements: all of these application scenarios will bring important benefits to humanity; at the same time, they also imply radical impact and need for rapid adjustment in our ways of living, as well as in our educational, social and political systems. Future-readiness is crucial and yet very challenging to achieve.

New fundamental questions will need to find answers without undue delay. What will be the meaning of privacy and intimacy in the Post-Information society? How will it be possible to ensure that the increased productivity of our interconnected systems goes for the benefit of a growing majority and not for a restricted part of the population? How will society facilitate growth and well-being while fighting against those who intend to make malevolent use of powerful and yet invisible technologies? And, coming closer to the individual: What will be the optimal collaboration point between the human biological brain and the various forms of artificial intelligence surrounding it? How will intelligence be defined and measured in this environment? How will personality, perception, cognition, and emotions be transformed when we will constantly live surrounded by hybrid physical, augmented, and virtual environments? What will be the overall impact of these new technologies on well-being, development, education, psycho-pathologies and their related therapies? Will life be extended well beyond one hundred years, and what will it mean to live a happy life then?

Indeed too many interesting questions, that cannot even be approached in the small playground of an editorial paper. Exciting research lies ahead of us, that should be fueled by appropriate funding. Let’s only focus our attention on the first critical issue we discussed: the role of humans in the Post-Information Society’s working eco-system, an environment shared with robotic as well as non-anthropomorphic artificial labor, and let’s try to draw the consequences on the emerging role of psychology, in collaboration with other disciplines. It is already clear that in the short term the probability of a specific job to be shifted from humans to computers is inversely proportional to its intrinsic creativity content (Bakhshi, Frey, & Osborne, 2015). But what is at stake in the medium-long term is the meaning, if any will be left, of human employment itself. In fact, any form of routine work, or in the limit any sort of employee position, will progressively lose its appeal or sense for humans in the future. These tasks will much more efficiently be carried out by the artificial labor force. Then, unless one believes that most human beings will simply not work and sustain themselves and their families with a guaranteed minimum salary, it can be argued that the new form of social justice will entail *the possibility for everyone to exploit artificial labor* in an entrepreneurial fashion, albeit possibly on a very small scale. In other words, in the biological-cyber mind collaboration, our organic creativity-driven role will be to generate surprising ideas, and then carry them on to realization to produce tangible or intangible goods or services, according to business models that will have extremely rapid lifespans. No one should be denied the right to take advantage of artificially intelligent systems. Possibly this will become a constitutional principle. But then, our very existence, and not only our dignity, will be related one-to-one with our ability to generate ideas with a potential for originality and effectiveness, i.e. with our creativity, to successfully catch the opportunities that technology is offering us. Hence the responsibility to study, understand, develop organic creativity as the discipline leading to productive survival, dignity, and well-being becomes more a sort of multidisciplinary mission, with psychology as its core engine.

In conclusion, the Post-Information Society is rapidly coming of age, and there is no way to slow down this transition. I argue that organic creativity will be essential for our survival in the necessary collaboration between humans and machines. We need to evolve our science of the human with large investments in research, and psychologists should be at the center of a multidisciplinary collaborative effort including sociologists, economists, computer scientists, engineers and all other relevant disciplines to establish creativity as a scientific discipline in order to produce necessary radical changes in our behaviors and enable new forms of long-lived well-being.
